# Precision microbiota therapy for IBD: premise and promise

**DOI:** 10.1080/19490976.2025.2489067

**Published:** 2025-04-07

**Authors:** Manabu Nagayama, Lasha Gogokhia, Randy S. Longman

**Affiliations:** a Jill Roberts Institute for Research in Inflammatory Bowel Disease, Weill Cornell Medicine, New York, NY, USA; b Jill Roberts Center for Inflammatory Bowel Disease, Division of Gastroenterology and Hepatology, Department of Medicine, Weill Cornell Medicine, New York, NY, USA

**Keywords:** Inflammatory bowel disease, Crohn’s disease, ulcerative colitis, precision microbiome therapeutics

## Abstract

Inflammatory Bowel Disease (IBD) is a spectrum of chronic inflammatory diseases of the intestine that includes subtypes of ulcerative colitis (UC) and Crohn’s Disease (CD) and currently has no cure. While IBD results from a complex interplay between genetic, environmental, and immunological factors, sequencing advances over the last 10–15 years revealed signature changes in gut microbiota that contribute to the pathogenesis of IBD. These findings highlight IBD as a disease target for microbiome-based therapies, with the potential to treat the underlying microbial pathogenesis and provide adjuvant therapy to the emerging spectrum of advanced therapies for IBD. Building on the success of fecal microbiota transplantation (FMT) for *Clostridioides difficile* infection, therapies targeting gut microbiota have emerged as promising approaches for treating IBD; however, unique aspects of IBD pathogenesis highlight the need for more precision in the approach to microbiome therapeutics that leverage aspects of recipient and donor selection, diet and xenobiotics, and strain-specific interactions to enhance the efficacy and safety of IBD therapy. This review focuses on both pre-clinical and clinical studies that support the premise for microbial therapeutics for IBD and aims to provide a framework for the development of precision microbiome therapeutics to optimize clinical outcomes for patients with IBD.

## Introduction

Although the underlying cause of inflammatory bowel disease (IBD) is not known, intestinal inflammation is thought to result from an interaction between altered gut microbiota and host genetic susceptibility.^1^ Early studies in Crohn’s disease (CD) revealed the expansion of enteric pathobionts using culture- and PCR-based technologies, providing early credence to the existence of gut dysbiosis in IBD.^2^ Small clinical studies supporting the use of antimicrobial therapy for the treatment of CD-associated ileitis bolstered the hypothesis that gut microbiota played a causal role in intestinal inflammation.^3–5^ Consistent with a causative role for gut microbiota, seminal work showed the requirement for the fecal stream in driving recurrent CD post-operatively.^6,7^ Although antimicrobial therapy is not effective for luminal inflammation in ulcerative colitis (UC), early studies with probiotic therapies showing a benefit in UC patients with mild to moderate disease that are incompletely controlled on mesalamine engendered significant enthusiasm for microbiome-based therapies, especially given the large proportion of UC patients with these clinical characteristics.^8^ With the emergence of sequencing technologies over the past two decades, many studies now corroborate early findings of microbial gut dysbiosis associated with new-onset disease.^9^ The more recent studies provide greater taxonomic resolution, which highlights the potential role for specific gut pathobionts (commensal bacteria with potential to exacerbate inflammation) in IBD. In parallel, sequencing advances which led to the mapping of the human genome and the discovery of genetic polymorphisms in IBD also identified numerous genetic variants contributing to dysregulation of host-microbe interactions in subjects with IBD.^1^ Meanwhile, advances in microbiome-based therapy led by the clinical success of fecal microbiota transplantation (FMT) for *Clostridioides difficile* (previously known as *Clostridium difficile)* infection provided a framework for shaping microbial-based therapies for IBD. Despite the success of early FMT studies for IBD, variability in results and trial heterogeneity have limited the overall impact of this therapy in shaping practical management of IBD, and it remains a knowledge gap.^10,11^ This review will explore the lessons learned from clinical trials of FMT for IBD as well as the premise, conceptual framework, and the major gaps in knowledge that still exist in the implementation of precision microbiota therapy approach for IBD.

## FMT for IBD: lessons learned for guiding a precision microbiome approach

Variations of FMT have been used in clinical medicine for centuries to treat diarrheal diseases. Early anecdotal reports of the safety and efficacy of FMT for pseudomembranous colitis led to initial case reports highlighting the potential efficacy for the emerging epidemic of recurrent *C. difficile* infection (CDI).^12^ With the subsequent demonstration of the efficacy in randomized control trials,^13^ FMT has served as the workhorse for clinical studies providing practical advancements in our understanding of microbial therapy. The overwhelming success of FMT for recurrent CDI and recent approvals of commercial microbial therapies (characterized as live biotherapeutic products or LBPs) have paved the way for their study in IBD. However, despite the thematic overlap of intestinal symptoms and inflammation, the disease pathologies underlying CDI and IBD are mechanistically very different. CDI is a bacterial infection and mechanisms of colonization resistance likely drive the efficacy of FMT and LBPs in preventing recurrent CDI. In contrast, IBD is not an infection which can be mitigated by colonization resistance, but rather a complex interaction between diverse microbiota and host susceptibility. As such, despite their clinical success and approval for recurrent CDI, several LBPs have failed to meet primary endpoints in UC, even with rigorous evidence documenting the kinetics of strain-level engraftment.^14^ These findings likely reflect the fact that the functional impact of different strains (and not just engraftment) may be critical for mediating the impact of LBPs in inflammatory disease.

**Figure 1.**
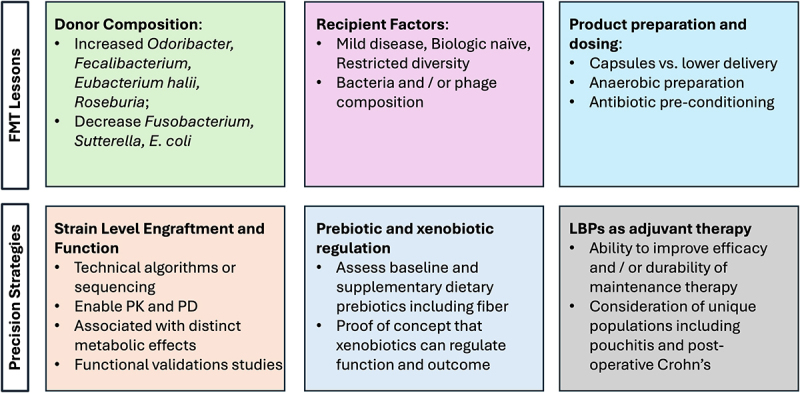
Lessons learned from FMT studies and strategic approach for precision microbiome therapy for IBD.

Over the last 10 years, several RCTs have studied the efficacy of FMT for IBD. Most of these studies focus on UC and only one RCT is available for CD evaluating the impact on maintenance of remission. A recent comprehensive meta-analysis of 12 studies with a total of 550 participants found increased rates of clinical remission in UC subjects that received FMT compared to control treatments.^15^ The risk ratio for induction of clinical remission was 1.79 (95% confidence interval: 1.13 to 2.84), indicating a significant benefit of FMT for mild to moderate UC. While the specifics of the clinical outcomes of these studies are summarized by others,^16^ the collective findings underscore the need for further research to better understand the long-term safety and efficacy of this novel approach. Despite the promising potential benefit of microbial therapy for UC, most of these studies are small cohorts and are complicated by heterogenous study designs. The overall effect size is likely overestimated and much larger studies would be needed to rigorously evaluate standard endpoints. However, given the need for standardization of production and enhanced safety, rationally designed LBPs will play a critical role in guiding future approaches. Several key factors may account for the variability in FMT study outcomes, and the insights learned from these early studies can help guide a more precision approach for rationally designed LBPs for IBD in the future (Figure 1).

### Donor variability and composition

A main source of the heterogeneity in clinical response to FMT for UC likely rests in donor variability. While FMT therapy from a diverse group of healthy donors is uniformly sufficient for providing colonization resistance required to treat CDI, the difference in the both underlying pathophysiology and mechanism of host-microbe interactions of IBD likely underlies the variable efficacy of donors seen in multiple studies.^17–20^ Several studies support the findings that a specific composition of strains or group of bacteria in the transferred product impacts clinical outcome.^21,22^ Consistent with this impact of donor variability, some studies failed to demonstrate the efficacy of FMT for UC. In one study using single donor FMT which failed to demonstrate efficacy for UC, 4 of the 8 donors accounted for all 6 subjects achieving clinical remission, whereas 4 donors used in 25 recipients (out of 51) failed to achieve even a single clinical remission.^19^ Another recent study which even attempted to evaluate this donor dependence based on rigorous donor selection including density normalization and selection of a “non-Bact2” composition, which was previously associated with active disease, also failed to demonstrate clinical remission in line with previous results.^20^ In this study, 15 donors were used for 26 recipients making it difficult to discern donor differences, but, of the 3 remissions achieved, 2 were from a donor used in 5 donations (40% remission) and 1 was from a donor used for 2 donations (50% remission). Thus, despite attempts for rigorous donor selection, the criteria needed to optimize selection of a “superdonor” still remain elusive.

To try to address this issue and increase the possibility of identifying the “good” donor strains, studies utilizing pooled donors show that they can maximize the potential donor impact.^23,24^ Pooling strategies overall seem to increase efficacy,^25^ however this approach also limits the ability to identify donor differences responsible for therapeutic efficacy. A two-donor strategy was able to identify donor-specific effects while maintaining overall clinical efficacy in an open label trial.^18^ Metagenomic investigation of the microbiome identified several taxa associated with clinical response including *Odoribacter, Fecalibacterium, E. hallii, Roseburia inulivorans* and the absence of pathobionts including *Fusobacterium, Sutterella* and *E. coli* species.^21,22^ Supporting the sufficiency of microbiota from a “good” donor to induce clinical remission, a lyophilized product from these donors was also able to achieve clinical remission.^26^ These encouraging results support the premise for a transition to a more refined LBP product to overcome the issue of donor variability and enhance efficacy. Investigational studies of LBPs derived from bacteria cultivated from successful donors identified a collection of potential bacteria targets including *Odoribacter splanchnicus* ^22^ and *F. prausnitzii*.^27^ Rationally designed consortiums of LBPs including these targets and/or the metabolic effector functions associated with response achieved impressive results in humanized mouse model of colitis,^28^ but additional studies are needed to assess their sufficiency in IBD.

### Recipient factors

The baseline microbiome of the recipient, disease severity, and concomitant medications can influence the response to FMT. Patients with more severe dysbiosis or refractory disease may respond differently compared to those with less severe conditions. Post-hoc analysis of UC responders to FMT revealed an association with characteristics of mild disease severity, left sided disease, lower fecal calprotectin, and biologic/advanced therapy naïve recipients.^17,18,29^ It remains unclear if this defines a microbiologically distinct subset of patient conditioned for response or simply a less severe spectrum of disease. UC subjects achieving clinical remission with FMT had a higher fecal species richness at baseline which was maintained during and after FMT compared to those that did not.^21^ Algorithms designed to highlight strain-level engraftment further indicate that engraftment was more successful in patients with reduced diversity.^30,31^ While this may simply signify a deficiency in the recipient that can serve as a stratification tool, the composition of recipient microbiota may actively shape engraftment through mechanisms of strain exclusion or IgA specificity.^32,33^ Furthermore, little is known about the potential role for recipient bacteriophage populations on the efficacy of FMT and shaping strain engraftment.^34^ Future studies should help to clarify if specific LBP-host pairing is required for shaping engraftment and efficacy.

### FMT protocols

Variations in FMT administration protocols, including the route of delivery (e.g., colonoscopy, enema, oral capsules), frequency of administration, preparation methods (anaerobic vs. aerobic vs. washed microbiota),^23,35^ and antibiotic pre-conditioning, still need clarification. Studies using colonoscopic delivery of FMT preparations were more successful in terms of clinical efficacy, but similar remission rates were achieved with either intensive, repeated, or even just single colonoscopic delivery.^17,18,23,24^ Although initial success with FMT required only bowel preparation, highlighting the potential role for UC dysbiosis itself in enabling a receptive environment, it is not clear if this will be sufficient for a per oral route of administration, which would be logistically preferable for microbial-based therapy. For CDI therapy, per rectal FMT or oral capsules are superior to nasogastric tube administration, although differences in strain-level engraftment between colonoscopy and oral capsule delivery still need to be defined.^36,37^

The use of antibiotic pre-conditioning has also been an area of debate. Given the initial findings of specific taxa associated with a reduced efficacy of FMT for UC including *Fusobacterium*, *Sutterella* and *E. coli* species, antibiotic pre-conditioning to mitigate these potentially inhospitable species and clear space for LBP engraftment was used successfully in the LOTUS trial.^26^ Similarly, although Phase 2 studies using the SER-287, a spore-based LBP, failed to show clinical efficacy for UC, pre-conditioning with vancomycin promoted strain-level engraftment.^14^ A related consideration is the bacterial density of the FMT or LBP, which can also play a role in regulating host physiology.^38^ Anaerobic preparation methods, which better preserve microbial viability, have shown promising results, but recent efforts to ensure high density in donors revealed that it alone was not sufficient for re-capturing donor efficacy.^20^ As the field moves toward rationally designed and manufactured LBPs, detailed characterization using traditional metrics of pharmacokinetics and pharmacodynamics should guide product density and dosing.

## IBD pathobionts as a therapeutic target for precision microbiome therapy

**Figure 2.**
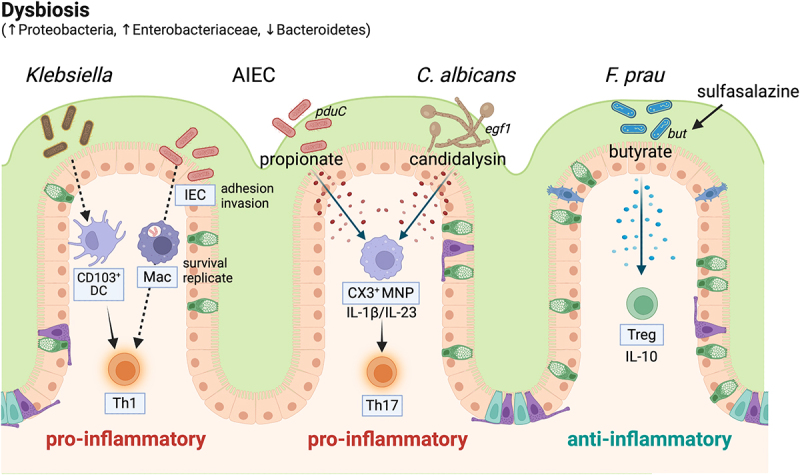
Key features of host-microbe interactions underlying the premise of microbial-based therapy for IBD.

The gut microbiome, consisting of trillions of bacteria, viruses, fungi, and other microorganisms, coats the surface of the GI tract. The microbiota is essential for providing nutrients and vitamins critical for maintaining gut health, highlighting the microbiome as a functional organ in human physiology. Given the proximity of the microbiome to the inflamed mucosal surface present in IBD, dysbiosis – or the imbalance in the microbiome – commonly observed in IBD is believed to contribute to disease pathogenesis.^39^ Studies have shown that IBD patients often exhibit reduced microbial diversity and an altered composition of gut bacteria compared to healthy individuals.^9,40,41^ This guilt-by-association is bolstered by genetic evidence highlighting IBD risk polymorphisms in microbial receptors that likely interact with microbial products.^1^ In contrast to initial clinical studies of bulk FMT blocking recurrent infections by colonization resistance, the collective data in IBD studies highlight a roadmap for a more precision approach to microbiome therapeutics. Several themes could guide this approach (Figure 2), including: a) the expansion of pathobionts that could serve as therapeutic targets; b) the disappearance of “beneficial bacteria” and their production of key metabolites that could be repleted by probiotics or functionally enhanced with xenobiotics; c) the contribution of microbes other than bacteria including fungal species and bacteriophages; d) a specific role for keystone species or sentinel bacteria in driving immune cell activation.

### AIEC as a model for targeting pathobionts

Despite the clinical of success of FMT studies in subjects with UC, microbial dysbiosis is more frequently associated with CD and is frequently characterized by the expansion of Enterobacteriacea.^42^ As an example of a pathobiont associated with IBD, the frequent isolation of *E. coli* adherent to the ileal mucosa in subjects with CD led to the categorization of a specific pathotype called Adherent-Invasive *E. coli* (AIEC).^43–45^ While AIEC defy a uniform genetic characterization and span traditional distinct phylotypes, they conform to a functional definition including epithelial cell adherence, epithelial cell invasion, survival and replication in macrophage, and the lack of invasive virulence factors. Pre-clinical studies support a pathogenic and potential metabolic role for AIEC in driving intestinal inflammation.^46–48^ Given the collective evidence for AIEC as a pathobiont, at least during times of active inflammation in CD, therapeutic strategies have been developed to target AIEC colonization. For example, synthetic molecules mimicking the carbohydrate recognition domain of the bacterial adhesins on the surface of AIEC that enable colonization reflect an anti-adhesive strategy to selectivity target AIEC.^49^ AIEC-specific bacteriophages can also serve as a therapeutic strategy to specifically target and kill AIEC to reduce colonization. Pre-clinical studies using AIEC-specific bacteriophages show decrease AIEC colonization and reduce colitis in mouse models, which support ongoing clinical studies evaluating the potential efficacy in CD.^50^ A Phase 1/2a study to assess the safety and efficacy of *E. coli* phages called EcoActive that target AIEC in subjects with inactive CD is ongoing (NCT03808103). Although AIEC is one of the more common pathobionts in CD, other species primarily within the Enterobacteriaceae including *Klebsiella pneumoniae* (Kp) have been identified as pathobionts in pre-clinical models. Seminal first-in-human studies of Kp specific phages showing efficient engraftment highlight the potential for specific Kp targeting strategies as microbial-based therapy in IBD.^51^ Adding to this approach, recent studies report the capability of targeted gene editing of *E. coli* and *K. pneumonia* strains directly in situ.^52^ These advances illustrate strategies for precise targeting of pathobionts with the potential for personalized therapy.

### Beneficial bacteria and the production of metabolites

In addition to the expansion of specific pathobionts, IBD-associated dysbiosis is marked by the depletion of commensals that are critical for promoting microbial homeostasis and the production of anti-inflammatory metabolites.^53^ Short-chain fatty acids (SCFA), in particular, play an important role in promoting epithelial cell homeostasis via multiple mechanisms,^54^ but their impact in clinical studies is still emerging. Surprisingly, anaerobically prepared samples did not increase fecal SCFA in UC subjects after receiving FMT,^23^ but it is not clear if local uptake or metagenomic capacity was changed.^55^ Several taxa may play critical roles in the production of SCFA and have the potential to be regulated by diet and medicines. Clostridial clusters of bacteria IV/IVXa including *F. prausnitzii* are major producers of butyrate regulated by the enzyme butyryl-CoA:acetate-CoA-transferase (But).^56^ *F. prausnitzii* is reduced in patients with active disease and is a negative predictor of ileal recurrence of CD post-operatively.^57^ In addition to its metabolic contribution, the beneficial impact of *F. prausnitzii* may result from the production of other anti-inflammatory molecules.^27^ Therapeutic studies are currently underway to assess the efficacy of *Fecalbacterium* strains as LBPs for IBD.

Sentinel bacterial species also contribute to production of a wide range of bioactive molecules including polyamines, sphingolipids, and bile acid metabolites.^54^ While changes in bile acids in IBD may result from a combination of defects in bile acid malabsorption coupled with defects in bile acid metabolism, seminal studies have identified key constituents of the microbiome that regulate these small molecule effectors.^58–60^ Recent technical advances enable the genetic engineering of a wide range of gut bacteria, providing a new platform to evaluate the functional impact of strains within a complex microbial environment.^61^ The ability to engineer native bacteria with transgenes to provide stable gain-of-function is currently employed by several ongoing clinical trials (including some for IBD)^62^ and provides transformative potential for microbial therapeutic approaches in IBD.^63,64^

### Transkingdom interactions

Given the limitations in sequencing technology and data repositories available for alignment, less is known about the non-bacterial constituents of IBD microbiota. For example, although fungal species are only 1–2% of the overall gut biomass, several studies highlight a role for the mycobiome in shaping mucosal and systemic immunity in IBD. Early clinical data identified increased antibodies to *S. cerevisiae* mannan (called anti-*S. cerevisiae* antibodies or ASCA) in patients with CD and the potential utility of these antibodies in prognostically identifying patients that will develop disease. However, the role for ASCA and fungal species generally in pathogenesis of IBD is only still emerging. Most of the clinical therapeutic studies of fungi in IBD have focused on *S. boulardii*, yielding variable clinical data which have done little to impact clinical practice or treatment guidelines. While there is pre-clinical data for *S. boulardii* protection through interaction with *Enterobacteriace* to promote epithelial barrier health, further studies are required to define the potential clinical utility of these isolates. *Candida albicans* and *C. parapsilosis* are consistently increased in fecal or mucosal samples from subjects with CD, but changes in *C. tropicalis, Saccharomyces* and *Malassezia spp* are variable.^65,66^ The expansion of *C. albicans* may be promoted by cross-feeding with bacterial constituents including the expansion of *E. coli* and other *Enterobacteriacea*e.^67^ Recent studies revealed the potential impact of both species-specific factors and hyphae formation in modulating colonization and mucosal immunity.^68,69^ Although initial pilot studies suggested the potential clinical benefit of fluconazole in reducing post-operative recurrence,^70^ follow up studies did not reveal a clinical benefit of anti-fungal therapy despite the overall reduction in *C. albicans* associated with disease activity reduction.^71^ It is possible that more subtle features of the mucosal immune response may dictate the response to fungi in IBD. For example, the cytokine IL-23 is very important for organizing anti-fungal immunity. Clinical strategies targeting fungal elements that are refractory to conventional treatment with IL-23 blockade therapy may help to guide a role for adjuvant precision anti-fungal therapy for IBD.

In addition to fungi, viruses and bacteriophages constitute a significant fraction of the microbial biomass. Like the gut bacteria, enteroviruses can provide critical signals to induce homeostatic mucosal immunity as well as modulate the severity of intestinal inflammation in susceptible hosts.^72,73^ Shifts in viral populations, particularly in bacteriophage communities, are implicated in the pathophysiology of IBD. Recent evidence highlights that bacteriophage, especially members of the Caudovirales family, are enriched in both CD and UC patients compared to healthy controls.^74^ However, the taxonomic specificity of this enrichment remains poorly characterized. An important distinction lies in the proportion of lytic versus temperate bacteriophage populations in IBD versus healthy individuals. In healthy individuals, lytic bacteriophages predominate, contributing to the maintenance of microbial diversity through predation of bacterial hosts.^75^ In contrast, individuals with IBD show an increase in temperate virions, which may promote microbial dysbiosis by integrating into host bacterial genomes, altering bacterial community dynamics, and inducing prophage activation under inflammatory conditions.^76^ This shift from lytic to temperate bacteriophage virions in IBD could exacerbate inflammation by driving bacterial blooms and releasing phage-associated products that interact with the immune system. Recent studies reveal that bacteriophage can be detected directly by the immune system and this may contribute to the potential effects of filtered product in fecal transplants.^77^ These insights are particularly relevant for microbial-based IBD therapies. For example, temperate phage reduction or modulation may serve as a target to restore microbial homeostasis and limit inflammation, while the immune recognition of phage-associated products might offer opportunities for immunomodulatory interventions. Additionally, the presence of certain phages might prevent successful engraftment of beneficial bacteria during FMT and contribute to the failure of a microbial therapy. Thus, while both enteroviruses and bacteriophages are critical to the IBD landscape, the Caudovirales family and the temperate-lytic phage dynamic represent key, underexplored areas with direct implications for emerging therapeutic approaches.

### Sentinel microbes regulate gut immunity

Seminal studies using gnotobiotic mouse models revealed that specific types of bacteria play key roles in shaping mucosal immunity, including *Bacteroides fragilis* ,^78,79^ Segmented Filamentous Bacterium (SFB),^80,81^ and species from Clostridium cluster IV and XIVa.^82^ Adherent bacteria such as SFB also induce robust production of mucosal immunoglobulin A (IgA), which is critical for host protection.^83^ IgA maintains intestinal homeostasis by forming a protective mucosal barrier, neutralizing pathogens and toxins, regulating gut microbiota composition, and facilitating engraftment of commensal bacteria.^33^ SFB also induces mucosal T cells that produce the cytokine IL-17 (called Th17 cells). Th17 cells are important in protecting the host from infectious challenges;^81^ however, under inflammatory conditions, these cells can also be responsible for fueling inflammation.^84^ Analogous to these effects of SFB, the adherent and metabolic characteristics of AIEC derived from subjects with CD promote the induction of inflammatory Th17 cells.^48^

Inflammatory T cells in the intestine can be controlled by a key subset of regulatory T cells in the intestine. Screening of gut-derived strains enabled the discovery of key human gut-derived bacteria capable of driving intestinal regulatory T cells.^85^ Strain-specific effects support the role for defined microbiota transplants in restoring T cell immune balance and serving as the premise for early-stage clinical development.^86^ Gnotobiotic mouse models have similarly been employed to identify and isolate immunoglobin (Ig) reactive bacterial strains that can shape the intestinal immune response.^87^ Isolation and characterization of bacteria bound to Ig identifies sentinel transferable strains capable of inducing mucosal immunity in the recipient that could help to drive precision microbiome therapies.^22,88^ Strain-specific effectors differentially regulate the induction of regulatory T cell subsets in vivo^89^ and recent studies have identified mechanistic elements of these strains which are stable over time.^90^ Seminal work showing the induction of regulatory T cells by Clostridium cluster IV and XIVa bacteria led to the development of LBPs currently in early-stage clinical trials for the treatment of UC (NCT05370885). The continued identification of strain-specific immune responses will help shape the design of precision microbial therapy for IBD.

## Precision microbiota therapy for IBD: concepts and future approach

Emerging from the concepts provided by pre-clinical studies and guided by the practical insights from FMT, precision microbiota therapy represents an evolution in therapeutically targeting the microbiome to treat IBD. The goal of this approach is to leverage culture and sequencing insights to define a product with composition and function that is effective for the treatment of IBD. Despite the early success of FMT in UC, a precision microbiota composition would hold benefits of reproducibility, allowing formal metrics to assess pharmacokinetics and pharmacodynamics, and increasing safety. Microbiota-based therapies are unlikely to be a one size fits all model. It is likely that therapies effective for CD will differ from those for UC, and it would be ideal for clinicians to be able to tailor treatment to target the underlying microbial dysbiosis. This would involve being able to define microbial-based metrics associated with success (e.g. engraftment), tailor therapy for disease type (e.g. patient stratification), integrate microbial therapy with other advanced therapies (e.g. combination therapy), and leverage diet and medicines including prebiotics and xenobiotics to optimize outcomes.

### Measuring strain-level engraftment, metabolic function and immunity

Even with the knowledge of donor composition, strain-level engraftment remains a challenge.^37^ Strain tracking algorithms have emerged but still rely on the ability to determine and track genetic variants.^30,31^ New technological advances will aid in this discovery including Microbe-seq technologies capable of single genome resolution within a complex microbial community.^91^ Even if one can define the features of a “strain” genetically, emerging evidence suggests that functional characterization of the strain *in situ* is required to understand the potential immune cell impact.^90^ Precision approaches to microbial therapy will need to leverage these strain tracking technologies to provide a proximal assessment of the microbial pharmacokinetics that can guide clinical decision making.

In addition to tracking pharmacokinetics of engraftment, metabolomics can be used as a proxy for the characterization of pharmacodynamics of microbial drug products interacting with their targets. Several studies showing a distinct metabolome exists following FMT^21,54^ highlight several classes of molecules that need better definition. The production of SCFAs including butyrate is a desired effect of microbial therapies based on many pre-clinical studies, but the impact of FMT on SCFA abundance post-transplant in subjects with UC is variable.^21,23^ In addition, the central role for bile acid metabolism in mucosal homeostasis highlights these pathways as a proximal target of precision approaches. Specific bacterial strains can play an essential role in generating isomers of secondary bile acids that can shape mucosal T cell polarization.^58,60^ Many of the enzymes responsible for these metabolic targets remain poorly characterized. New technologies are still emerging to help define these metabolic functions *in situ*. For example, activity-based probes have emerged to fill this niche by mapping the enzymatic function even in complex microbial communities.^92^ This functional characterization has the potential to help map more defined metabolic targets that could amplify the efficacy of emerging therapies. In addition, these technologies offer the possibility to isolate microbes directly based on enzymatic activity or metabolic function, providing a new approach for culturing strains for future therapies.^93^

### Leveraging prebiotic and xenobiotic regulation to optimize microbial-based therapy for IBD

The importance of considering the impact of diet in guiding microbial-based therapy is clear.^94^ Seminal studies showing the impact of milk fats in expanding pathobionts that can exacerbate colitis in genetically susceptible backgrounds reveal the need to consider diet in the design of microbial-based therapies for IBD.^95^ This impact of diet can have rapid effects on the composition of the microbiome.^96^ The interaction of the microbiome with the diet is dynamic, highlighted by seminal studies showing the role for antibiotics in the efficacy of interventions to treat malnutrition.^97^ Cultural impacts of diet can have a significant effect on the microbiome composition and function, and this should be considered in trial design and data analysis of precision microbial therapy in geographically and culturally diverse cohorts.^98^

Fiber is a key prebiotic component of the diet responsible for modifying microbiota composition as well regulating the production of downstream metabolites including SCFAs in both health and disease.^99,100^ Since dietary fibers are not hydrolyzed by mammalian enzymes but instead utilized by bacteria in the GI tract, it has been suggested that this can serve as a prebiotic to help shape engraftment and function. The beneficial effects of combining FMT with dietary fiber are supported by placebo-controlled Phase 2 trial testing the role for prebiotic fiber supplementation with oral encapsulated FMT in a population of Americans with obesity and metabolic syndrome.^101^ Recipients with higher microbial diversity and dietary fiber intake at baseline exhibited better engraftment and metabolic outcomes following FMT and fiber supplementation.^102^ Although initial guidelines for IBD sought to reduce fiber intake,^103^ leading to inadequate levels of fiber intake in subjects with IBD,^104,105^ studies revealing an association of UC with lower abundance of beneficial metabolites associated with fiber including SCFAs^106^ led to a “re-thinking” of this dietary approach.^107^ Although dietary fiber is complex and the quantification in clinical trials is challenging, future clinical trials can evaluate the impact of specific supplemental fiber formulations in shaping the efficacy of microbial therapy for IBD.

In addition to fiber, case reports combining FMT with a specific diet begin to offer clinical data supporting a broader role for dietary prebiotics. Diets high in fermentable fibers and low in sulfur-containing proteins led to sustained remission in a UC patient and highlight the need for studies in IBD.^108^ The FMT-AID study from India validated the role for an anti-inflammatory diet (which includes high levels of dietary fiber) in combination with FMT as effective therapy to induce and maintain remission for over 1 year in subjects with mild to moderate UC.^29^ Additional studies are needed to both define the impact of baseline diet as well as interventional prebiotics coupled with microbiome analysis to tailor precision microbiota therapy, enabling more consistent and sustained therapeutic outcomes in subjects with IBD.

In addition to prebiotics and dietary supplements, xenobiotics (or non-endogenous chemical substances including medicines) can also be leveraged to regulate the function of the microbiota. The features of this are nicely illustrated by early seminal work showing the role for the microbiome in metabolizing the cardiac glycoside Digoxin.^109,110^ Recent work revealing the role for *Eggerthella lenta* as the critical commensal regulating Digoxin metabolism also highlighted the impact of dietary arginine in regulating the transcription of the enzyme responsible for its metabolism.^111^ A related example of xenobiotic regulation of the microbiome in IBD is the functional regulation shown by sulfa drugs. Subtherapeutic levels of sulfamethoxazole are sufficient to block folate synthesis leading to an upregulation of colipterins that promote the production of anti-inflammatory molecules in *E. coli*, suggesting that, in addition to the antibacterial function, this xenobiotic can proactively stimulate bacteria to reduce colitis.^112^ In addition, antagonism of folate synthesis by sulfapyridine can augment butyrate production by inducing transcription of the main butyryl transferase in *F. prausnitzii*, shunting production of acetate to butyrate.^113^ First-in-human studies are needed to test if these xenobiotics can promote function of microbiota *in situ* to optimize microbial-based therapy for diverse cohorts with diverse diets.

### Microbial-based therapy as an adjuvant for advanced IBD therapy

The optimal timing for precision microbiota therapy within the disease course remains unclear. For FMT in UC, the majority of the studies focus on induction of remission, but an equally important endpoint is the maintenance of remission. Unfortunately, combination of budesonide with FMT did not potentiate either clinical efficacy or strain-level engraftment.^114^ A major unmet need in IBD remains the treatment ceiling limiting induction of remission and separately the durability of most therapy at 1 year. Combination therapy of FMT with biologic therapy is under study to see if it can improve durability at 1 year and remains a fertile space for microbial-based therapy research in IBD.

While most studies of FMT and microbial-based therapy studies focus on the treatment of UC, additional subsets of IBD may benefit from microbial-based therapy. Pouchitis is known to be a microbe responsive inflammatory pathology and frequently responds to antibiotic therapy; however, refractory pouchitis remains a clinical dilemma. Early studies suggested the role for VSL#3 in the prevention and treatment of pouchitis, but the real-world efficacy for this therapy has been variable.^11,115,116^ In addition, both post-operative CD and fibrotic disease remain open areas ripe for investigation. Early work suggested that the ileal microbiome correlated with the risk of post-operative recurrence.^57^ Post-operative therapy with metronidazole is used in high-risk cases to decrease the risk of recurrence. Targeted study design and patient stratification is a major opportunity for microbial-based therapy for IBD. Finally, there are currently no therapies that exist to treat fibrotic disease in CD. Recent data suggest that some of the key pathologic findings in CD including creeping fat result from a response to sealing off bacterial penetration.^117^ Further studies are needed to understand microbial-based therapy strategies to reduce transmural and fibrotic complications in CD.

### Challenges and future directions

Precision microbiota therapy represents a paradigm shift in the management of IBD, offering the potential for personalized treatments with efficacy, safety, and durability. By leveraging the unique microbiome profiles of patients, this approach aims to restore gut health and achieve sustained remission. The promising results from clinical trials and case studies underscore the importance of continued research and development in this field. As our understanding of the gut microbiome and its role in IBD deepens, precision microbiota therapy is poised to become a cornerstone of personalized medicine in gastroenterology.

Implementing microbial-based therapy presents several challenges and limitations. Technical challenges include accurately analyzing and interpreting microbiome data to identify relevant microbial targets. Methodological challenges involve designing robust clinical trials that account for the variability in individual microbiomes. Ethical and regulatory issues must also be considered, including the development and use of genetically modified organisms. Given the genetic and environmental differences of subjects from different geographic and cultural backgrounds,^118^ it is important for future studies to capture data from diverse cohorts.

Safety is a key issue in developing an impactful microbiome-based therapy. Although conceptual concerns of the transfer of pathogenic bacteria in FMT have been largely mitigated by rigorous screening, rational design of microbial consortia will further reduce safety concerns. However, the impact of these strains, particularly in subjects with a compromised mucosal barrier and frequently on immunosuppressive therapy, will need to be monitored. Despite these challenges, the future of microbial-based therapy holds great promise. Advances in metagenomics, bioinformatics, and personalized medicine are likely to drive further innovations to improve the safety and specificity of this therapeutic modality. Continued research and collaboration among scientists, clinicians, and regulatory bodies will be essential to realize the full potential of this therapeutic approach for IBD.

## Data Availability

The data that support the findings of this study are openly available and provided in the References section below.
